# O02 Impact of dietary habits on AMR development in human gut bacteria: insights from a hollow fibre infection model

**DOI:** 10.1093/jacamr/dlad143.002

**Published:** 2024-01-03

**Authors:** Jegak Seo, Frank Kloprogge, Andrew Smith, Lena Ciric

**Affiliations:** Civil, Environmental, and Geomatic Engineering, University College London, Gower Street, London WC1E 6BT, UK; Institute for Global Health, University College London, Royal Free Campus, Rowland Hill Street, London NW3 2PF, UK; Eastman Dental Institute, University College London, Royal Free Campus, Rowland Hill Street, London NW3 2PF, UK; Civil, Environmental, and Geomatic Engineering, University College London, Gower Street, London WC1E 6BT, UK

## Abstract

**Background:**

As efforts to reduce antibiotic exposure through medical prescriptions have made significant progress, the potential consequences of residual antibiotics in animal-based food sources have been somewhat overlooked. The question of whether chronic exposure to antibiotic remnants in human diets could lead to the development of antimicrobial resistance (AMR) in human gut bacteria remains largely unanswered. This research aimed to address this gap by utilizing the hollow fibre infection model (HFIM) to investigate whether routine consumption of antibiotics from an omnivorous diet contributes to the emergence of resistance in a previously antibiotic-susceptible strain of *E. coli* isolated from the human gut.

**Methods:**

To select the antibiotic mixtures for our study, we conducted a comprehensive diet survey involving 131 participants. Additionally, we employed LC-MS to quantify antibiotic concentrations in the most commonly consumed animal-based food products. We rigorously analysed triplicates of 34 food and beverage items, encompassing beef, chicken, pork, fish, and dairy, to detect and measure the concentrations of 10 specific antibiotics. Our findings revealed the presence of low levels of nine antibiotics across the samples, with amoxicillin and trimethoprim being the most frequently detected antibiotics. Alarmingly, 12 out of 34 products exceeded the acceptable daily intake levels for amoxicillin, ampicillin, and enrofloxacin. We further adapted the estimated daily intake formula to calculate antibiotic combinations and their intake from each meal. Subsequently, we subjected bacterial cells to the HFIM for 7 days, exposing them to three antibiotic ‘meals’ daily. We collected samples from the HFIM every 24 h to monitor changes in resistance phenotypes through MIC testing, as well as for subsequent transcriptomic analysis.

**Results:**

Astonishingly, we observed the development of resistance to both individual antibiotics and combinations of antibiotics from the very first day, with resistance levels progressively increasing over the course of the week. Interestingly, the rate at which MICs increased was most rapid in the initial days, followed by a slower rate of increase while MICs continued to rise. This study provides compelling evidence that chronic exposure to low levels of antibiotics, akin to those found in animal-derived foods, can lead to rapid resistance development across multiple antibiotics within human gut bacteria. Further examination of samples from the HFIM through RT-PCR and WGS is warranted to elucidate the mechanisms underlying resistance development in the gut environment due to dietary factors.

**Conclusions:**

Our research highlights the urgent need for greater scrutiny of antibiotic residues in the food chain and emphasizes the potential role of dietary habits in driving AMR development within human gut bacteria. Understanding the underlying mechanisms is crucial for devising effective strategies to mitigate this emerging public health concern.

